# Supplementary material for: Assess the efficacy of China’s Inter-provincial Government Services policy: A quantitative evaluation based on PMC-Index model
Source: PLoS One. 2024 Dec 12;19(12):e0310491. doi: 10.1371/journal.pone.0310491 (PMC11637331; doi:10.1371/journal.pone.0310491)
Supplement: S1 File — (DOCX) [file pone.0310491.s001.docx]

The policy documents used in the manuscript are publicly available and are linked below:

State Council:

<https://www.gov.cn/zhengce/zhengceku/2020-09/29/content_5548125.htm?ivk_sa=1023197a&sid_for_share=80113_2>

Chongqing:

<http://wap.cq.gov.cn/ykbzt/yhyshj/zcjj/zz/202011/t20201118_8614437.html?sid_for_share=80113_2>

Gansu:

<https://zwfw.gansu.gov.cn/minle/xwfb/swszfapbs/zcwj/art/2022/art_dc8fcda1c2c9412089e20d7373b53c8d.html?sid_for_share=80113_2>

Guizhou:

<http://www.guizhou.gov.cn/zwgk/zcfg/szfwj/qfbf/202011/t20201126_70473714.html?sid_for_share=80113_2>

Tianjin:

<https://www.tj.gov.cn/zwgk/szfwj/tjsrmzfbgt/202011/t20201127_4139578.html?sid_for_share=80113_2>

Guangdong:

<http://www.gd.gov.cn/zwgk/gongbao/2020/34/content/mpost_3367022.html?sid_for_share=80113_2>

Yunnan:

<https://www.yn.gov.cn/zwgk/zcwj/yzfb/202011/t20201127_213808.html?sid_for_share=80113_2>

Fujian:

<http://www.fujian.gov.cn/bsfw/bmfw/zcwj/202012/t20201207_5476051.htm?sid_for_share=80113_2>

Jiangxi:

<https://www.jiangxi.gov.cn/art/2020/12/9/art_4975_2962674.html?sid_for_share=80113_2>

Hebei:

<http://www.hebei.gov.cn/columns/3d33a20b-4271-4b3b-8cae-3664e980d262/202010/26/fbddf3b6-4f8b-11ee-beb8-6018954d7f6f.html?sid_for_share=80113_2>

Sichuan:

<https://www.sc.gov.cn/10462/zfwjts/2020/12/14/39adb164e0e64b3b8825830276ed145f.shtml?sid_for_share=80113_2>

Xinjiang:

<https://www.xinjiang.gov.cn/xinjiang/gfxwj/202102/5c4ac6b6e49b4afba11ecbac2d9fefa6.shtml?sid_for_share=80113_2>

Inner Mongolia:

<https://www.nmg.gov.cn/zwgk/zfxxgk/zfxxgkml/202012/t20201214_366071.html?sid_for_share=80113_2>

Jiangsu:

<http://www.jiangsu.gov.cn/art/2020/12/28/art_46144_9617062.html?sid_for_share=80113_2>

Zhejiang:

<https://www.zj.gov.cn/art/2020/12/29/art_1229620653_2402608.html?sid_for_share=80113_2>

Hubei:

<http://www.hubei.gov.cn/zfwj/ezbf/202012/t20201231_3185777.shtml?sid_for_share=80113_2>

Jilin:

<http://xxgk.jl.gov.cn/szf/gkml/202101/t20210104_7883463.html?sid_for_share=80113_2>

Anhui:

<https://www.ah.gov.cn/szf/zfgb/553956051.html?sid_for_share=80113_2>

Hunan:

<http://www.hunan.gov.cn/hnszf/szf/hnzb_18/2021/202101/szfbgtwj_98720_88_1qqcuhkgvehermhkrr/202101/t20210119_14143156.html?sid_for_share=80113_2>

Guangxi:

<http://www.gxzf.gov.cn/zfwj/zxwj/t8322258.shtml?sid_for_share=80113_2>

Shandong:

<http://www.shandong.gov.cn/art/2021/4/1/art_107861_111219.html?sid_for_share=80113_2>

Tibet:

<https://www.xizang.gov.cn/zwgk/zfxxgk/fdzdgknr/zc/xzgfxwj/202212/W020221202382415634286.pdf>

Shanxi:

<http://www.shanxi.gov.cn/zfxxgk/zfcbw/zfgb2/2021nzfgb_76606/d8q_76614/szfbgtwj_77923/202205/t20220513_5978671.shtml?sid_for_share=80113_2>

Henan:

<http://m.henan.gov.cn/2021/08-06/2197328.html?sid_for_share=80113_2>

Liaoning:

<https://ysj.ln.gov.cn/ysj/zfxxgk/zfxxgkzd/lzyj/bbmgfxwj/D480E1CF33FD439AB697579295D35EAE/?sid_for_share=80113_2>

Heilongjiang:

<https://www.hlj.gov.cn/hlj/c107892/202208/c00_31378115.shtml?sid_for_share=80113_2>

Qinghai:

<http://swt.qinghai.gov.cn/zf/fd/zc/gz/202211/t20221123_191425_wap.html?sid_for_share=80113_2>

Beijing:

<https://www.beijing.gov.cn/zhengce/zhengcefagui/202401/t20240102_3522031.html?sid_for_share=80113_2>
