# Supplementary material for: Assess the efficacy of China’s Inter-provincial Government Services policy: A quantitative evaluation based on PMC-Index model
Source: PLoS One. 2024 Dec 12;19(12):e0310491. doi: 10.1371/journal.pone.0310491 (PMC11637331; doi:10.1371/journal.pone.0310491)
Supplement: Attachment — Submitted filename: detailed response to reviewers.docx [file pone.0310491.s002.docx]

Dear editors and reviewers:

We thank you very much for your helpful and insightful comments and suggestions on our manuscript entitled “Assess the efficacy of China’s Inter-provincial Government Services Policy: A Quantitative Evaluation Based on PMC-Index Model” .These comments and suggestions are very helpful for improving our paper. We studied them carefully and made some revisions according to the comments and suggestions of the associate editor and the reviewers. The main changes in the paper are marked in red color and the responses to the comments are as follows.

**Reviewer #1**: ABSTRACT
*The abstract effectively introduces a study evaluating China's Inter-provincial Government Services Policy using a quantitative approach based on the PMC model. It succinctly presents the findings regarding policy design, consistency, and identified weaknesses, along with proposed optimization recommendations. However, it could benefit from clearer language and organization to enhance readability and impact. For instance, the author could consider restructuring the abstract to begin with a concise statement of the research aim or problem addressed by the Inter-provincial Government Services Policy. Then, provide a brief overview of the methodology employed, followed by a clear presentation of the key findings and their implications. Additionally, using simpler language and avoiding jargon can improve accessibility for readers unfamiliar with the PMC model or policy evaluation terminology.*

**Response:** We are very grateful to the reviewer for the positive comments. Based on the reviewers' suggestions, this paper has restructured the abstract. First, the author added a concise statement of the research aim at the beginning. Next, the author provided a brief overview of the methodology employed, followed by a clear presentation of the key findings and implications. At the same time, we have revised the paper in more concise language according to your request. All changes are shown in red. **(See Abstract, Page 1)**.

***INTRODUCTION***

*The introduction could benefit from clearer organization and language to improve readability. For instance, dividing the introduction into subsections such as Background, Significance of the Policy, Research Objective, and Highlights of the Paper would provide clearer structure. Using more concise language to convey key points would enhance readability and make the introduction more engaging.*

**Response:** Thank you for your comments. Based on your suggestions, we have rearranged the introduction into background, policy implications, research objectives, and highlights. All changes are reflected in red. **(See Introduction, Page 1-Page 3)**.

***LITERATURE REVIEW***

*The literature review provides a comprehensive overview of existing research on the Inter-provincial Government Services Policy and policy evaluation methods. To enhance clarity and readability, consider implementing the following suggestions:

a) The author can introduce clear subheadings for each subsection of the literature review to help readers navigate through different topics more easily. For example, the author could use subheadings like "Perspectives on Inter-provincial Government Services Policy" and "Methods of Policy Evaluation."
Recommendation:
Perspectives on Inter-provincial Government Services Policy
Collaborative Governance
Digital Transformation
Holistic Theoretical Approaches
b) The author needs to ensure that the language used is clear and accessible to readers from diverse backgrounds. Avoid overly technical terms or jargon unless it necessary. Recommendation:
Instead of: "The endogenous endowment of 'public-technical governance-overlapping action' of cross-domain governance of government services is refined by some scholars, who put forward three practice modes of cross-domain governance of government services: vertically embedded, internally originated hair style, and governance of government and society (enterprise), and constructed the development path of cross-domain governance of government services in the four dimensions of strategy, supply and demand, technology, and ecology development path."
Use: "Scholars refine the concept of 'public-technical governance-overlapping action' and propose three practice modes for cross-domain governance of government services: vertically embedded, internally originated, and governance of government and society."*

**Response:** We really appreciate the valuable advice. Firstly, we have added subheadings for the first subsection of the literature review. Secondly, we have trimmed and modified some of the statements to make them easier for readers of different backgrounds to understand. And the content is marked in red color. **(See Section 2.1, Page 3- Page 4)**

***RESEARCH DESIGN*** *a)The section lacks clear subheadings to delineate different aspects of the research design. Adding subsection headings such as "Research Sample and Methodology," "Policy Feature Recognition," "Construction of PMC Index Model," and "PMC Surface Drawing" would enhance readability and organization. Recommendation:
Before: "3.1 Research Sample and Methodology"
After: "3.1 Research Sample and Methodology: Selection Criteria and Data Collection"*

*b) The text contains lengthy explanations and repetitive phrases, which could be streamlined for brevity. For instance, instead of repeating phrases like "the high frequency of terms such as," the author could use more concise language to convey the same meaning. Recommendation:
Before: "From the perspective of policy guarantee mechanism, terms such as 'integration, management, acceptance, lead, docking coordination, support, guidance, coordination, supervision, and reliance' appear more frequently, indicating that accelerating the construction of Inter-provincial Government Services requires a variety of ways of coordination..."
After: "Policy guarantee mechanisms, including integration, management, acceptance, and coordination, are crucial for accelerating the construction of Inter-provincial Government Services."*

*c) The author needs to ensure smooth transition sentences between paragraphs can improve the flow of ideas. Connecting sentences that link each step of the research design process would provide readers with a clearer understanding of the methodology's progression. Recommendation:
Before: "3.2 Policy Feature Recognition. On the basis of obtaining the policy text..."
After: "3.2 Policy Feature Recognition: Following the acquisition of policy texts, the next step involves..."*

*d) Some sentences may be difficult for readers to comprehend. Simplifying complex phrases and using straightforward language would enhance clarity. Recommendation:
Before: "The visualization results show that keywords 'government', 'services', 'departments', 'sharing' and 'platforms' have a high centrality."
After: "The visualization results indicate that certain keywords, such as 'government' and 'services', hold significant centrality within the semantic network."*

*e) While the text references tables and figures, it would be beneficial to integrate them more seamlessly into the narrative. Directly referring to specific figures in the text and explaining their relevance to the discussion would enhance understanding. Recommendation:
Before: "The PMC surface figure is a symmetric surface based on the values of nine first-level indexes, which can show the pros and disadvantages of various policies, so as to better evaluate Inter-provincial Government Services policies formulated by the Chinese government."
After: "The PMC surface figure (Figure 1) displays a symmetric surface representing the values of nine first-level indexes, providing insights into the strengths and weaknesses of Inter-provincial Government Services policies."*

**Response:** Thanks for the valuable suggestions. We have made changes to the corresponding sections according to your request, as follows: First, we modified the subheadings utilized in the research design section to make it clearer what follows.**(See Section 3.1 and 3.2, Page 5 and Page 7)**.Second, we have deleted lengthy explanations and repetitive phrases, and replaced them with more concise expressions **(See Section 3.2, Page 7)**.Next, in section 3.1 and 3.2, we have added transitional sentences between paragraphs to make the process of research design clearer to the readers **(See Section 3.1 and Section 3.2,Page 5 and Page 7).**Additionally, we've simplified complex phrases and used straightforward language for greater clarity **(See Section 3.2,Page 9).** Finally, we make direct references to specific charts in the text and explain their relevance to the discussion, allowing the table to fit more seamlessly into the narrative **(See Section 3.3.4, Page 12)**. Specific changes can be found in red in the paper.

***RESULT AND COMPARATION ANALYSIS*** *a)The section title "Results and Comparative Analysis" could be clearer if it explicitly mentions what is being analyzed. Also, consider breaking down the content into smaller subsections for easier navigation and comprehension. Recommendation:
Before: "4. Results and Comparation analysis"
After: "4. Evaluation and Comparative Analysis of Inter-provincial Government Services Policies"
b) Some sentences contain redundant phrases or repetitive information. Try to convey the same meaning with fewer words to improve readability and efficiency. Recommendation:
Before: "From the calculation results of the PMC index of the 28 policies (see Table 6), the PMC index scores range from [6.45 to 8.62], with a mean value of 7.71, and 15, 16, and 19 are acceptable levels, while the rest of the policies are excellent levels, and there are no bad and perfect policies. perfect level policies."
After: "The PMC index scores of the 28 policies ranged from 6.45 to 8.62, with a mean value of 7.71. Notably, policies 15, 16, and 19 attained acceptable levels, while the others achieved excellence."
c)The author need to introduce smoother transitions between subsections to improve the flow of the analysis. Clear transitions help guide the reader through the logical progression of ideas. Recommendation:
Before: "4.2.1 Characterization of overall policies"
After: "4.2.1 Overview of Policies: Analyzing Overall Characteristics"
d) The author needs to simplify complex sentences and technical language to ensure clarity and accessibility for all readers. Recommendation:
Before: "Comparing the scores of each level of policy variables with the mean value, it is found that..."
After: "Upon comparing the scores of each policy variable with the mean value, it becomes evident that..."*

*e) The author needs to ensure that references to figures are clear and seamlessly integrated into the text. Figures should complement the analysis and be introduced in a way that enhances understanding. Recommendation:
Before: "In order to visualize the results of PMC index calculation, the first-level index scores of each policy can be transformed into a third-order matrix as shown in Eq.(5), and then the internal consistency level of each policy can be analyzed by observing the concavity and convexity of the surface."
After: "To visually represent the PMC index calculation results, we transform the first-level index scores of each policy into a third-order matrix (Eq.5), facilitating analysis of each policy's internal consistency level through surface concavity and convexity."*

**Response:** Thanks for these helpful suggestions. We've made changes to the content in line with the detailed suggestions you have given. First, we revised the title of Section 4 and subdivided the content of the title of 4.2.1 into smaller subsections so that it clearly mentions what is being analyzed.**(See Section 4 and Section 4.2.1,Page 12 and Page 14)**.Second, we eliminated sentences containing redundant phrases or repetitive information to convey the same meaning in fewer words.**(See Section 4.2.1 and Section 4.3.1,Page 14 and Page 15)**.After that, we revised the title of 4.2.1 to allow for smoother transitions between subsections so that the reader understands the logical progression of ideas.**(See Section 4.2.1,Page 14)**. Then, we simplified complex sentences and technical language to ensure clarity and accessibility for all readers **(See Section 4.3.1, Page 15)**.Finally, We modified the expression of the sentences to ensure that references to figures are clear and seamlessly integrated into the text.**(See Section 4.2.2, Page 14)**.All changes are highlighted in red.

***CONCLUSION*** *The author needs to:
(a) Divide the conclusion into smaller paragraphs focusing on specific aspects, such as findings, recommendations, and future research directions.
(b) Ensure consistent use of terminology, such as "Inter-provincial Government Services Policy," throughout the conclusion.
(c) Refer to relevant figures, such as the PMC index calculation results, to support statements about policy effectiveness or areas for improvement.*

**Response:** Thanks for the valuable suggestion. (a) We have revised the conclusion of the paper according to your requirements, and marked the corresponding revisions in red, which part of the article is specified. (b) We have modified some expressions to ensure that terminology is used consistently throughout the conclusions. (c) In the conclusion, we cite figures to support statements about the effectiveness of the policy or areas for improvement. **(See Section 5.1, Page 18-Page 20)**.The changes have been highlighted in red.

***Reviewer #2:*** *1. In the literature review, the contents of Section 2.1 are not strongly related to its title, Research on Inter-provincial Government Services Policy. The authors mainly illustrate collaborative governance, digital transformation, and holistic governance, with limited research reviews on Inter-provincial Government Services Policy.*

**Response:** We greatly appreciate that the reviewer has pointed this out. We have added a synthesis of some studies on Inter-provincial Government Services Policy and made appropriate changes and adjustments to the content.**(See Section 2,Page 3-Page 4)**.

*2. What is the significance of the keyword network analysis in Section 3.2? How does it contribute to the construction of the PMC index model? Please make the rationale clearer.*

**Response:** According to the reviewer’s suggestion, we added the significance of keyword network analysis and showed how it can help in constructing PMC index models. **(See section 3.2,Page 9)**.

*3. In Section 3.3.4, PMC Surface Drawing, the text mentions three figures (Figures 2-4), but only Figure 2 is seen at the end of the paper. Please confirm whether the uploaded images are correct.*

**Response:** Please forgive us for leaving out two pictures. We have added two figures to the document.**(See Figure 2 and Figure 3)**.

4. *In the conclusion section, the author states that the main body of policy formulation is the government, and that experts, think tanks, and research institutions are not involved in policy formulation. How was this conclusion reached? How did the author obtain information on the composition of policy participants from text analysis and the PMC index model?*

**Response:** Thanks for pointing out the issues. We are very sorry not to explain how the conclusions were reached, but in fact we have come to the composition of policy actors through statistical analysis, i.e., the main body of policy-making is the government, experts, think tanks and research institutions. Among them, experts, think tanks and research institutions are less involved in policy- making. To avoid ambiguity, we have removed the corresponding elements from the conclusions and reorganized them to make them more revealing.**(See Section 5.1,Page 19)**.

5. *The article lacks discussions on the limitations.*

**Response:** Thanks for the valuable suggestion. We add a discussion of limitations at the end of the article.**(See Section 5.2,Page 20).**

*6. The study uses the PMC index model to evaluate China’s Inter-provincial Government Service Policy, but the theoretical contribution and practical significance of the study are not well embodied.*

**Response:** Thank you for your constructive comments. We apologize for not clarifying the theoretical contribution and practical implications of this article. We have rearranged the article, and the theoretical contributions and practical significance of this paper are as follows:(1)Theoretical Contributions: The first is the innovation of policy evaluation methods. This paper proposes a policy evaluation method based on the PMC model, which provides a new perspective for understanding the effect of policies. Through the quantitative study of the policy text, the lack of evaluation methods of the existing cross-provincial general policy is supplemented. With the help of the PMC index model, the PMC surface diagram is combined with the depression index to analyze the characteristics and shortcomings of the existing policies. This opens up new avenues for public policy evaluation. The second is to deepen the theory of policy evaluation. From the perspective of policy formulation, this paper conducts a quantitative text analysis of the inter-provincial general policy, conducts policy evaluation, and explains the effectiveness and consistency of policy implementation. (2)Practical implications: First, quantitatively evaluate the effect of the policy. The quantitative evaluation of China’s Inter-provincial Government Service Policy through the PMC index model can show the effect of policy implementation more intuitively. This approach can provide policymakers with strong data support. The second is to guide the direction of policy optimization. The PMC index model can not only evaluate the effectiveness of the strategy, but also visually show the strengths and weaknesses of each dimension of the strategy through the PMC surface diagram. There is still the possibility of policy optimization in terms of scope of application, timeliness and scientificity, which provides a clear direction for policy optimization for policymakers.

***Reviewer #3:*** *This paper applies the PMC Index Model to quantitatively assess inter-provincial government services policies in China to explore the effectiveness of policy implementation, which indeed is a beneficial research direction. The literature review is clearly structured, advances critical analysis of existing research, and achieves positive results in its conclusions.

The introduction directly presents the research question and clearly lists the contributions of the study. However, I believe that the choice of research methods should be briefly described at the beginning, highlighting the systematic, holistic, and predictive features and advantages of the policy text evaluation method to smoothly transition into the subsequent research.

The paper's conclusions focus on proposing policy optimizations, which need to be more grounded in the research data and results. I perceive some issues with parts of the conclusions where the derivations from the data and data analysis seem less validating than verifying, and the depth of the conclusions appears somewhat lacking. I recommend that the author:

Precisely align the logic connection between data analysis results and conclusions.
Strengthen causal inferences to ensure all conclusions and recommendations are directly based on data analysis results.
The manuscript is clearly written and meets the norms of English academic writing, but there are still some inaccuracies in grammar and word choice that might affect reader comprehension. Further polishing of the language is needed to meet the standards of academic publication.*

**Response:** We are very grateful to the reviewer for the positive comments. We really appreciate the valuable advice. **Firstly**, we supplement the introduction with a description of the choice of research methodology and highlight the characteristics and advantages of the policy text evaluation methodology in terms of its systematic, holistic, and predictive nature. **(See Introduction, Page 2)**. **Secondly**, in the conclusion section, we cited relevant figures to strengthen the logical link between the results of the data analysis and the conclusions. **Then**, we have revised the conclusions according to your requirements, elaborated on the research results, recommendations and future research methods, and further explained the reasonableness and depth of the conclusions. **(See Section 5.1, Page 19-Page 20). Finally**, we have invited professionals to linguistically polish the language and content of this article to ensure that it meets the standards for academic publication.
